# Adapting VMAT plans optimized for an HD120 MLC for delivery with a Millennium MLC

**DOI:** 10.1002/acm2.12134

**Published:** 2017-07-20

**Authors:** Samuel B. French, Stephen Bhagroo, Daryl P. Nazareth, Matthew B. Podgorsak

**Affiliations:** ^1^ Department of Radiation Medicine Roswell Park Cancer Institute Buffalo NY USA; ^2^ Medical Physics Program Jacobs School of Medicine and Biomedical Sciences University at Buffalo State University of New York Buffalo NY USA

**Keywords:** HD120, high‐definition MLC, linac transfer, Millennium MLC 120, patient treatment schedule, standard‐definition MLC

## Abstract

Linac downtime invariably impacts delivery of patients' scheduled treatments. Transferring a patient's treatment to an available linac is a common practice. Transferring a Volumetric Modulated Arc Therapy (VMAT) plan from a linac equipped with a standard‐definition MLC to one equipped with a higher definition MLC is practical and routine in clinics with multiple MLC‐equipped linacs. However, the reverse transfer presents a challenge because the high‐definition MLC aperture shapes must be adapted for delivery with the lower definition device. We have developed an efficient method to adapt VMAT plans originally designed for a high‐definition MLC to a standard‐definition MLC. We present the dosimetric results of our adaptation method for head‐and‐neck, brain, lung, and prostate VMAT plans. The delivery of the adapted plans was verified using standard phantom measurements.

## 1 INTRODUCTION

Ideally, a patient's entire treatment course is delivered in sequential daily fractions (weekends excepted) on the linear accelerator (linac) for which the radiation plan was designed; however, linac malfunction occasionally causes downtime, and may require the transfer of patient plans between linacs with differing multileaf collimator (MLC) designs. In our department, one linac is equipped with a Varian high‐definition MLC (HD120), while multiple linacs have standard‐definition Millennium MLCs (Millennium 120). All of these linacs are commissioned to deliver VMAT treatments. In addition to differences in leaf widths, the MLCs differ in material composition and geometric properties (leaf thickness, tongue‐and‐groove design, and leaf‐end curvature^1^, ^2^), which would lead to dosimetric differences between VMAT plans. Nevertheless, the single‐fraction transfer of a treatment may be desirable to maintain the prescribed fractionation schedule. Fractionation plays a sensitive and demonstrable role in patient outcomes for head‐and‐neck treatments,^3^, ^4^ and likely for other treatment sites.

A previous study^5^ investigated transferring VMAT patients between linacs using a Pinnacle‐based treatment planning system (TPS). Transferring a VMAT plan was not possible without reoptimization. With our method, a high‐definition VMAT plan (from a high‐definition linac, HDL) could be adapted to a standard‐definition linac (SDL) by creating a new plan (the “adapted” plan) using the DICOM plan file. The resulting adapted plan is analyzed within the TPS (Varian Eclipse 13.6) and exported to our record‐and‐verify system for treatment delivery.

Our goal with the plan adaptation system was to study a simple method that would allow for transferring of the VMAT patient from an HDL to an SDL.

## 2 METHODS AND MATERIALS

A MATLAB routine (MATLAB, R2007a, The MathWorks Inc., Natick, MA, USA) was written to copy VMAT delivery data (leaf aperture shapes, field weights and control point MU indices) contained in the planning DICOM file and place it into a prepared template. The template is a copy of the original plan which will act as a container for the transfer linac information. The template file differs from the original plan in the following way: the original linac is replaced by the transfer linac, and there are new dynamic MLC objects that are added by the user. The MLC objects must be added since the TPS automatically deletes the original MLC objects after a new linac is specified. The MATLAB code operates on two DICOM files: the original HDL plan, from which MLC positions are extracted, and the template, into which adaptations of the original MLC positions are placed. The VMAT plan information is extracted from the HDL DICOM into the MATLAB workspace, and the leaf shapes are averaged, or mapped one‐to‐one, to corresponding leaves in the standard‐definition SDL MLC, creating the adapted plan. The adapted plan is saved in the DICOM format and imported into the TPS (Varian Eclipse 13.6) where the full complement of dosimetry tools is available, for example, dose calculators, DVH displays, and plan summing. After dose calculation, we compared each patient plan between its original version and the adapted version where the prescription of the original plan was preserved, for example, 100% Rx dose to 95% of the target volume.

### 2.A MLCs and DICOM file creation

The Eclipse treatment planning system, version 13.6 (Varian Inc., Palo Alto, CA, USA), was used for this study. A copy of the original HDL plan was created in Eclipse and the linac was changed from the HDL to the SDL in the plan properties. This process automatically removes the dynamic MLC positions defining the control point apertures stored in the plan. A new MLC object was then added to each field (VMAT arc) in the SDL plan, and the number of control points was set by the user to match the original number found in the HDL fields. The TPS template method provides the beam modeling data required to calculate dose correctly for the SDL.

#### 2.A.1 Leaf adaptation: Mapping and averaging

**Figure 1:**
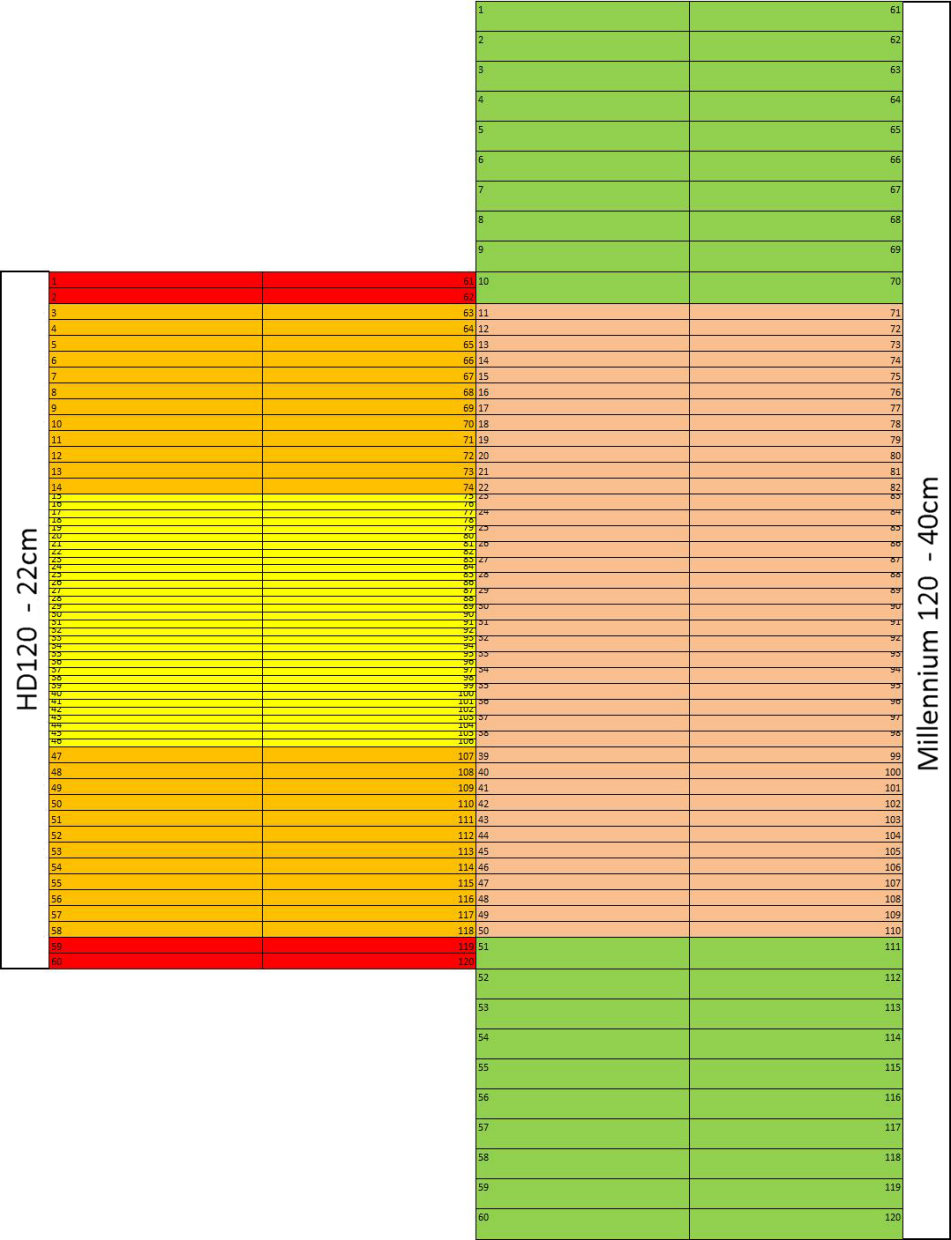
An illustration comparing the relative leaf widths and locations of the leaves between the HD120 MLC and the Millennium MLC 120.

**Figure 2:**
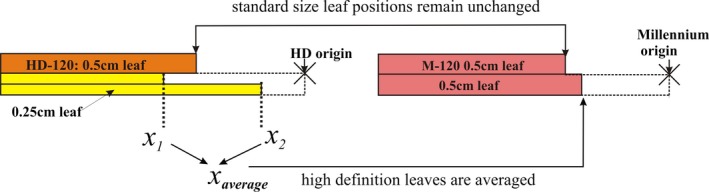
The method for adaptation of leaves. High‐definition leaves are averaged and assigned to single standard‐definition leaves. Leaves of similar width are mapped directly.

Both the HDL and SDL MLCs have 120 leaves (60 per bank). The HDL leaves have a 14–32–14 pattern of widths 0.5–0.25–0.5 cm, respectively. The SDL leaves have a 10–40–10 pattern of widths 1.0–0.5–1.0 cm, respectively, where all widths are measured at isocenter (Figs. 1 and 2).

**Figure 3:**
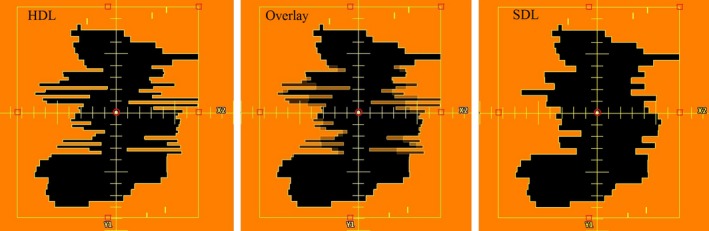
Images of a single control point from the original and adapted plans: The original (LEFT) and the adapted control point (RIGHT) show the effect of the averaging method in the central region (CENTER overlay).

Data objects extracted from the HDL plan include the positions of the MLC leaves and the control point MU meterset weights. The leaf positions determine the apertures at control points in the VMAT plan and the control point meterset weights determine the relative dose deposited by an individual control point within a VMAT arc. Figure 2 presents an illustration of the method by which leaves from the HDL are adapted to the SDL. High‐definition leaf positions are averaged two‐by‐two, and the resulting value is assigned to a corresponding leaf in the SDL MLC. Matched leaves of equal width have their positions mapped directly to the SDL MLC. A comparison of a single control point aperture is shown in Fig. 3. The original control point (Fig. 3, left) resembles its adapted counterpart (Fig. 3, right), being identical in the peripheral field, and displaying obvious changes to the central, high‐definition, region (Fig. 3, center overlay).

#### 2.A.2 Behavior of MLC leaf velocities under averaging

At the time that the original VMAT plan was created by the TPS, the optimization step included applying an MLC leaf‐speed constraint to the leaves that defined potential aperture sets. Let an individual leaf's maximum travel speed between adjacent control points be *C* (leaf‐speed constraint is the same for both linacs). A simple calculation shows that this constraint will also be satisfied by any leaf in the adapted plan (i.e., after the averaging process). Let $x_{1}$ and $x_{2}$ be the positions of two adjacent HDL leaves that are averaged to provide, $\vec{x}$, the position of an SDL leaf:

(1) $$\vec{x}=\frac{\vec{x_{1}}+\vec{x_{2}}}{2}.$$

The average velocity of this leaf is then,

(2) $$\vec{v}=\frac{d\vec{x}}{dt}=\frac{1}{2}\left(\frac{d\vec{x_{1}}}{dt}+\frac{d\vec{x_{2}}}{dt}\right)=\frac{\vec{v_{1}}+\vec{v_{2}}}{2}.$$

Since each HDL leaf obeys the maximum‐speed constraint,

(3) $$\left\{\begin{array}{c} \left|\vec{v_{1}}\right|<C\\ \left|\vec{v_{2}}\right|<C \end{array}\right.$$

and, by the triangle inequality,

(4) $$\left|\vec{v_{1}}+\vec{v_{2}}\right|\leq \left|\vec{v_{1}}\right|+\left|\vec{v_{2}}\right|.$$

Therefore,

(5) $$\left|\vec{v}\right|=\frac{1}{2}\left|\vec{v_{1}}+\vec{v_{2}}\right|\leq \frac{\left|\vec{v_{1}}\right|+\left|\vec{v_{2}}\right|}{2}<\frac{C+C}{2}=C,$$

which was the original SDL leaf maximum‐speed constraint.

## 3 RESULTS

### 3.A Changes to the dose to structures

The dose to the patient structures in the adapted SDL plans was calculated in the TPS using the Eclipse AAA algorithm and compared via DVH analysis to the corresponding structure doses in the original HDL plan. The percent difference between HDL and SDL doses (maximum and mean) to individual planning structures were calculated via

(6) $$\%diff=\frac{D_{SDL}-D_{HDL}}{D_{HDL}}$$

where $D_{SDL}$ is the mean or maximum dose to a structure in the SDL plan and $D_{HDL}$ is the corresponding dose in the original HDL plan. The homogeneity index, HI, was calculated for target structures using^6^

(7) $$HI=\frac{D_{MAX}-D_{MIN}}{D_{MEAN}}$$

Table 1 shows the mean percent difference (Eq. (6)) in maximum dose and mean dose between the HDL and the SDL planning structures for four general treatment sites (number of patients in parentheses): brain (13), head & neck (5), lung (4), and prostate (11).

**Table 1:** Mean percent differences in the target and organs‐at‐risk for four sets of treatment sites

Treatment site	Structure	Percent difference (mean ± st. dev.)
Brain	Brainstem mean	4.1 ± 5.3
	Brainstem max	7.0 ± 9.1
	Lens L mean	7.3 ± 8.7
	Lens L max	8.8 ± 10.9
	Lens R mean	6.6 ± 9.5
	Lens R max	6.1 ± 13.5
	Optic nerve L mean	4.0 ± 4.8
	Optic nerve L max	4.1 ± 4.8
	Optic nerve R mean	5.1 ± 16.3
	Optic nerve R max	7.1 ± 18.4
	Target mean	2.9 ± 2.3
	Target max	8.1 ± 3.7
Head & Neck	Brainstem mean	−2.6 ± 3.1
	Brainstem max	1.2 ± 3.0
	Parotid L mean	−0.7 ± 2.2
	Parotid L max	−0.9 ± 4.7
	Parotid R mean	−0.1 ± 2.2
	Parotid R max	−1.1 ± 4.8
	Spinal cord mean	2.1 ± 3.7
	Spinal cord max	5.8 ± 5.5
	Target mean	3.4 ± 2.4
	Target max	8.9 ± 4.9
Lung	Lung mean	−3.1 ± 3.2
	Lung max	2.8 ± 7.3
	Spinal cord mean	−8.3 ± 4.3
	Spinal cord max	−9.7 ± 3.3
	Target mean	−5.1 ± 4.2
	Target max	3.9 ± 5.4
Prostate	Bladder mean	2.5 ± 5.0
	Bladder max	8.0 ± 5.4
	Rectum mean	2.4 ± 3.7
	Rectum max	3.9 ± 3.5
	Fem head L mean	−0.4 ± 3.3
	Fem head L max	0.6 ± 5.1
	Fem head R mean	−0.7 ± 3.4
	Fem head R max	0.9 ± 4.3
	Target mean	−0.3 ± 3.4
	Target max	8.8 ± 5.3

We performed standard VMAT quality assurance measurements using the ArcCHECK^®^ device (Sun Nuclear, Melbourne, FL) to verify that the adapted plans were deliverable on the SDL. Verification plans were prepared for the ArcCHECK^®^ phantom in our TPS using the adapted SDL plans. Sun Nuclear SNCPatient^™^ software package where a distance‐to‐agreement (DTA) analysis was performed between the exported dose‐to‐phantom and the measured dose delivery on the SDL. All plans exceeded 93% Distance‐to‐Agreement analysis (98.4% ± 1.4%).

### 3.B Summation plan

**Figure 4:**
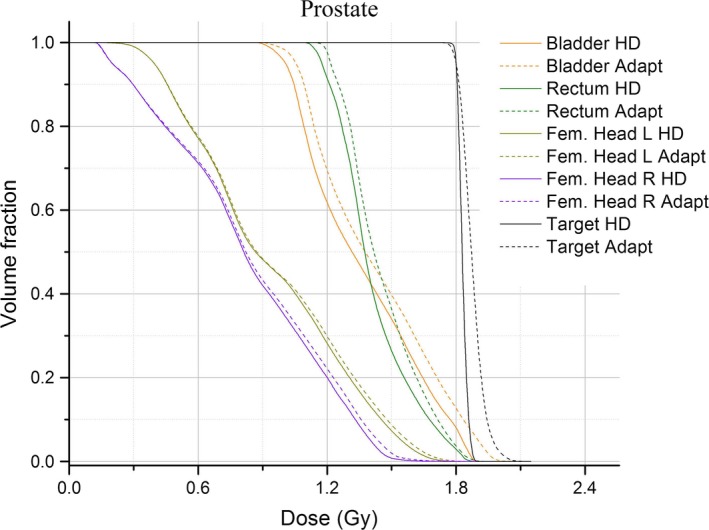
DVHs of one prostate patient. The original planned DVH's are solid; the adapted plan is shown with dashed lines.

**Figure 5:**
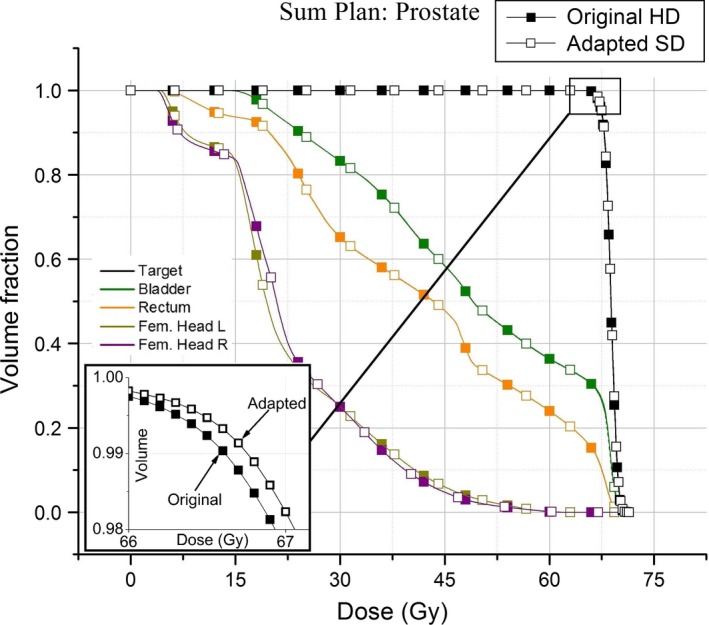
Plan sum for a prostate treatment. One fraction out of the entire course of treatment was adapted from the high‐definition linac to a standard‐definition linac.

The purpose of the adaptation method is to develop a deliverable plan for one fraction (Fig. 4) of a treatment course in order to maintain the patient's treatment schedule during linac downtime. The patient will most likely receive the remainder of their fractions of treatment on the original HDL machine. In a representative case a regular course of treatment of 1.8 Gy × 25 fractions followed by several boost courses had one fraction from the 25‐fraction course adapted and replaced in the summation plan (Fig. 5). The detail of the target structure's shoulder region shows a slight increase in the target structure's DVH in the adapted plan.

**Table 2:** Homogeneity indices of the original HD plans and their corresponding standard‐definition adapted plans

Treatment site	Structure	Percent difference (mean ± st. dev.)
Brain	Target HD plan	0.13 ± 0.11
	Target adapted	0.25 ± 0.09
Head & Neck	Target HD plan	0.10 ± 0.3
	Target adapted	0.21 ± 0.07
Lung	Target HD plan	0.11 ± 0.02
	Target adapted	0.27 ± 0.08
Prostate	Target HD plan	0.11 ± 0.02
	Target adapted	0.26 ± 0.05

### 3.C Clinical timeline

The intention of creating an adapted plan for an SDL is to allow a patient to continue treatment on the same day he/she was originally scheduled when the originally planned linac is down. The adapted plans need to be processed in the TPS and evaluated using DVH analysis and verified using phantom measurements in an efficient manner. We studied the time required to complete the adaptation tasks. We found that preparing an adapted fraction can be performed within an hour from end‐to‐end, that is, from the time that physics staff is notified of the desire to proceed with a treatment on a different linac to the evaluation of the verification plan delivery on the SDL. If an adapted plan is rejected for dosimetric reasons, the clinic pays a temporal cost of approximately 20 min since the plan will not be verified using phantom measurements.

**Figure 6:**
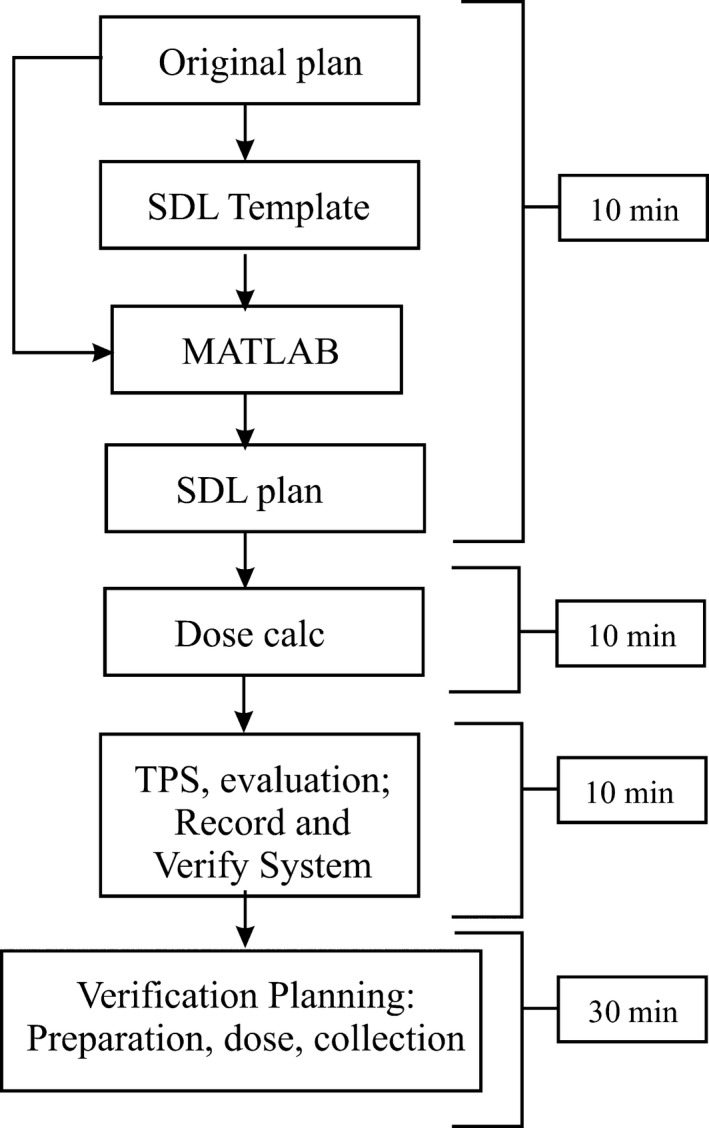
Clinical timeline for adapting a VMAT plan from the high‐definition linac to the standard‐definition linac. Dose calculation will vary for individual Eclipse users; the time quoted here is typical in our clinic.

The average time for individual tasks was quantified while adapting the set of HDL plans we used in our study. Most of the work involved the use of the TPS and was performed at a computing station with the exception of the delivery of the verification plans. The timeline in Fig. 6 outlines the general components involved in VMAT plan adaptation for a single patient with approximate times noted. There could be gains in speed due to tasks that are completed in parallel, that is, multiple physicists may work in tandem to process multiple patient plans.

## 4 DISCUSSION

### 4.A Dose changes

**Figure 7:**
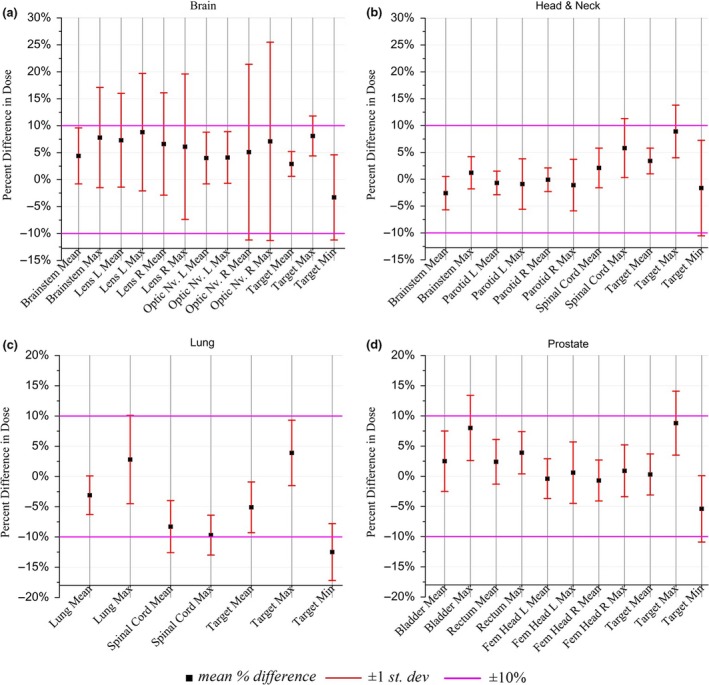
Mean percent difference between the original HDL and the plans adapted to the SDL for (a) brain, (b) head‐and‐neck, (c) lung, and (d) prostate.

**Figure 8:**
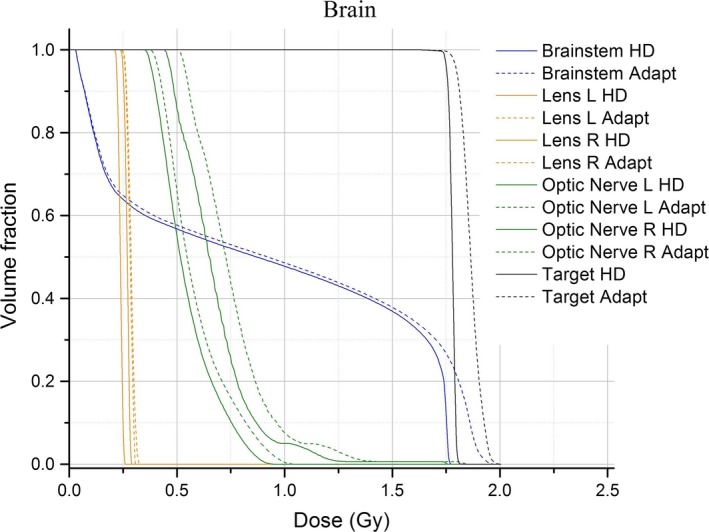
DVHs of one brain patient. The original planned DVH's are solid; the adapted plan is shown with dashed lines.

Changes in the maximum, mean, and minimum dose to structures between HDL and SDL plans were observed (Table 1). The mean doses to the structures both increased and decreased over a range (Fig. 7). The changes in DVHs of the target and OARS between the original and the adapted, single‐fraction, plans are observable (Figs. 4 and 8). There are a number of components of the MLC design, in addition to the leaf position changes, that lead to the dose changes observed. The differences in MLC designs include leaf profile geometry, material composition, and of course, the leaf widths being the most obvious difference. These differing attributes of the MLC designs must necessarily account for dosimetric differences between plans, and one design feature may dominate more than another in terms of its contribution to the dose changes. The uncoupling of these dose contributions is outside the scope of the present work, but we note that the following design features are most likely responsible for the changes observed in Tables 1 and 2.

The leaf ends have different radii of curvature with the high‐definition MLC having a 16 cm radius, and the standard‐definition MLC having an 8 cm curvature. The radii affect the radiation transport near the leaf ends, certainly, and it is possible that the effect of the leaf‐end curvature is a greater contributor to dose differences in the portions of the fields where no leaf averaging was performed. The changes to the leaf positions are another component of the observed dose differences and the averaging of the leaf positions in the high‐definition regions of the MLC probably contributes the greatest amount of dosimetric changes.

The effects for one patient treatment field (Fig. 4) displays the dose changes resulting from the adaptation method. Looking at the target DVH for an adapted plan, we observe an increase in the size of the tail region. This part of the target DVH characterizes a subset of the total voxels contained within the target structure. This voxel population receives more doses in the adapted plan than in the original. Since the adapted plan is normalized according to a prescribed dose in the same manner as the original HDL plan (typically, 100% Rx to 95% target volume), we can interpret the tail as the increase in the relative dose between the voxels represented by the tail portion to the remaining voxels in the structure.

**Figure 9:**
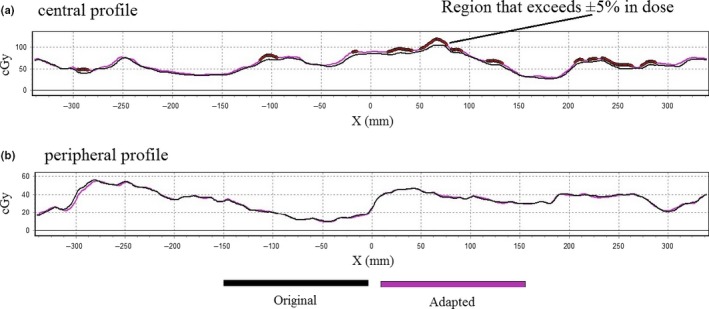
Dose profiles comparing an original prostate plan to an adapted plan. A profile from the central region (a), where leaf positions were averaged, displays more deviation between the plans. A peripheral profile (b), where leaf shapes are mapped between similar leaves, shows better agreement compared to the central region.

A profile of an adapted plan in the center of the field [Fig. 9(a)] shows dose deviations from the original plan (black line). The central region [Fig. 9(a)] shows the effects of leaf averaging as compared to a peripheral profile [Fig. 9(b)] where leaf‐mapping dominates.

The effect of the adapted fraction on the entire course of treatment is small (Fig. 5, detail) compared to the original HDL‐only dose (a complete course of treatment with no adapted fraction). Therefore, a patient functionally receives his/her full course of treatment with the incorporation of a single‐adapted fraction while maintaining the original schedule of treatment.

After discussing the results of our adaptation method, our clinical group has established a cutoff of ±10% in changes to the mean or max dose‐to‐structure. Structures that go outside this threshold will trigger additional review of the adapted DVHs. No review of the adapted treatment is required for plans where the 10% threshold is not violated by any structures. Figure 7 shows the mean percent difference for maximum and mean structure doses were ±10% for all structures analyzed. Increased dose to the target structures (Figs. 4 and 8) can be seen as well as rightward shifts in dose in the adapted plan.

Patient treatment outcomes are correlated with treatment schedules,^7^ and since the plan in summation is nearly equivalent to the original HDL‐only plan, we have assurance that we are treating the patient as if the originally planned linac was always available. This opens up the possibility of transferring the patient from an HDL to an SDL, thereby avoiding interruption to the patient treatment schedule and any indeterminate radiobiological effects due to the interruption of the fractionation schedule.

### 4.B Clinical cases

Prostate treatments were the best performers under adaptation. The mean dose to the rectum in prostate cases increased by 2.4 ± 3.7% with maximum target dose increasing 3.9 ± 3.5% in adapted plans showing that prostate tended to adapt well to the SDL adaptation vs the typical adapted brain plan. In brain cases, changes to optic nerve mean and maximum doses ranged much higher with adapted Optic Nerve R increasing 5.1 ± 16.3% and 7.1 ± 18.4% (mean and max, respectively). The spread in mean and maximum dose to the structures for prostate [Fig. 8(d)] is much smaller than for brain [Fig. 8(a)] over the set of all structures. This implies that the averaging technique used in our method leads to less degradation of the original prostate plan compared to the adapted prostate plan.

Head‐and‐neck (H&N) cases [Fig. 8(b)] performed well after adaptation with mean percent differences varying over a smaller range compared to Lung and Brain cases. Since the variance (red bars) extends over a smaller range compared to other treatment sites, a larger proportion of the population of adapted Head‐and‐neck plans will not violate the ±10% dose deviation policy, compared to brain and lung plans. This tighter variance demonstrates stability in adapted H&N VMAT plans that are especially apparent when comparing prostate to brain. Mean percent dose in prostate cases also showed similar stability to those of H&N. Lung cases [Fig. 8(c)] displayed better variance performance over the set of OARs relative to the brain structures, but less so compared to H&N and prostate.

### 4.C Clinical application and timing

Our clinic has a large proportion of VMAT case on our HDL. In a 1–2‐week period, 51.7% of the cases treated on the HDL were VMAT. During a period of HDL downtime, there could be more than 10 patients who are potential linac transfer candidates. Let one assume that replanning of the patient treatments for the SDL will take at least 2 h each. With two members of clinic staff working solely on the replanning tasks, verification planning deliveries would commence after at least 10 work hours. Considering our 10‐patient example, treatments would commence after an estimated 12.00 h (assuming two physicists performing verification planning and delivery at a cost of 25 min per patient plan). Our method allows the preparation of 10 adapted plans within 2 h (with two staff members) leading to a total of 5.42 h of time between the beginning of HDL‐to‐SDL adaptation until the completion of verification plan delivery.

## 5 CONCLUSION

We have developed an efficient method to adapt VMAT plans from HDLs to SDLs using a leaf‐position averaging process, and have shown the changes to patient dose distributions for a variety of treatment plans. Dose to structures in adapted plans were impacted as follows: the mean doses increased or decreased on the order of 1% while maximum doses increased on the order 10%. In our study of 33 cases, the DVH information indicates that clinically acceptable adapted plans can be produced for a single‐fraction treatment. However, since the change in mean or maximum doses to particular structures reaches >10% for some adapted plans, each plan should be evaluated individually before proceeding with treatment.

## CONFLICT OF INTEREST

The authors declare no conflict of interest.
